# Adaptation of Two Wild Bird-Origin H3N8 Avian Influenza Viruses to Mammalian Hosts

**DOI:** 10.3390/v14051097

**Published:** 2022-05-19

**Authors:** Jianpeng Liang, Qian Li, Linlin Cai, Qingli Yuan, Libin Chen, Qiuyan Lin, Chencheng Xiao, Bin Xiang, Tao Ren

**Affiliations:** 1 College of Veterinary Medicine, South China Agricultural University, Guangzhou 510642, China; ljp20171028006@126.com (J.L.); aqian6363@126.com (Q.L.); canlinlin2048@126.com (L.C.); yuanqingli1230@126.com (Q.Y.); chenlibin@scau.edu.cn (L.C.); linqiuyan@scau.edu.cn (Q.L.); 2 Key Laboratory of Animal Vaccine Development, Ministry of Agriculture, Guangzhou 510642, China; 3 National and Regional Joint Engineering Laboratory for Medicament of Zoonosis Prevention and Control, Guangzhou 510642, China; 4 Key Laboratory of Zoonosis Prevention and Control of Guangdong Province, Guangzhou 510642, China; 5 College of Animal Science and Technology, Shihezi University, Shihezi 832003, China; cc718@126.com; 6 Key Laboratory of Control and Prevention of Animal Disease of Xinjiang Production and Construction Corps, Shihezi 832003, China; 7 College of Veterinary Medicine, Yunnan Agricultural University, Kunming 650000, China

**Keywords:** H3N8 AIV, PA, PB2, mutation, cross-species transmission, mammal adaptability

## Abstract

Wild birds play an important role in the emergence, evolution, and spread of zoonotic avian influenza viruses (AIVs). However, there are few studies on the cross-species transmission of the H3N8 AIV originating from wild birds. In this study, we investigated the transmissibility and pathogenicity of two H3N8 low pathogenic avian influenza viruses (LPAIVs) isolated from wild birds, GZA1 and XJ47, to mammals. The *HA* genes of both strains belonged to Eurasian isolates, while the other genes were derived from a variety of other subtypes of AIVs. Both strains can infect specific-pathogen-free (SPF) chickens, BALB/c mice, and guinea pigs. The XJ47 strain spread horizontally in SPF chickens and guinea pigs. The GZA1 strain did not spread horizontally but caused higher weight loss and mild lung inflammation in mice. P12-GZA1- and P12-XJ47-adapted strains obtained after 12 passages in the lung of mice showed enhanced pathogenicity in mice, which led to obvious clinical symptoms, lung inflammation, and 100% death. Both adapted strains have the reported mutation T97I in the PA, and the reported mutation D701N in PB2 has been found in the P12-GZA1-adapted strain. This study provides an important scientific basis for the continuous monitoring of wild AIVs and the mechanism underlying AIV cross-species transmission.

## 1. Introduction

Avian influenza viruses (AIVs) are influenza A viruses (IAVs) belonging to the *Orthomyxoviridae* family [1]. Avian influenza is a poultry infectious disease with a high infection rate that causes great harm to the poultry industry. According to the classification of AIV pathogenicity by the World Organization for Animal Health, avian influenza is divided into low pathogenic avian influenza (LPAI) and highly pathogenic avian influenza (HPAI) [2]. IAVs are subtyped according to the antigenicity of the surface glycoproteins into 17 HA subtypes and 11 NA subtypes. Except for bat-derived influenza-like viruses H17N10 and H18N11 [3], all IAVs subtypes can be isolated from avian hosts.

In addition to wild birds and poultry [4], infected hosts of H3N8 IAVs include horses [5], dogs [6], cats [7], seals [8], and humans [9]. The H3N8 AIV causes almost no clinical symptoms in chickens after infection [4,10]. Segmented genomes from different influenza viruses can mix or recombine when the two viruses simultaneously infect the same host. In 2019, a new recombinant, H3N3 AIV, was isolated in South Korea, with PB2 and NS stemming from wild waterfowl, and the remaining six genes from poultry, indicating that wild birds and poultry exchanged internal fragments of the virus when living in common waters [11]. New recombinant AIV strains are emerging, indicating that it is necessary to continuously monitor the evolution of AIVs in wild birds [12].

At present, there is a large amount of research on the adaptation of AIVs to mammals. For example, Q226L and G228S mutations can increase the ability of AIVs to bind to the human sialic acid α-2,6 galactose receptor [13]. In H5 subtype AIVs, the mutations 101N and 160A of the HA protein can enhance the binding of the HA protein to the human-like receptor [14,15]. Previous studies have shown that a single amino acid mutation in the *PB2* gene of AIVs determines the host range [16]. Mutations in V292I, E158K, E627K, and D701N in the PB2 protein significantly enhanced polymerase activity, thereby promoting the ability of the mallard-origin-H2N2, mallard-origin-H4N6, duck-origin-H5N1, and birds-origin-H5N1 AIVs to cross the species barrier and infect mice or humans [16,17,18,19]. In addition, it has been reported that R or K at position 591 of PB2 can confer efficient viral replication in mammals [20]. Moreover, mutations N30D and T215A of M1 can enhance virulence in mice. Another mutation, P42S, in NS1 can also increase virulence in mice [21]. There are few studies on the pathogenicity of the H3N8 AIV. Only one related study found that the PB1 S524G mutation of the wild-bird-origin H3N8 AIV could enhance virulence and transmission in mammals [22].

Two H3N8 AIV strains were isolated in an epidemiological survey of wild-bird-origin AIVs from 2017 to 2020 along the East Asia-Australia and East Africa-West Asia migratory bird flyways. One strain was isolated from Gallinula in Guangzhou City, Guangdong Province, China, and designated as A/gallinula/Guangzhou/A1/2017(H3N8) (GZA1). The other strain XJ47 was isolated from a mallard duck in Xinjiang Province, China [23]. The current study helps deepen our understanding of the cross-species situation regarding wild-bird-origin H3N8 AIVs in China.

## 2. Materials and Methods

### 2.1. Viruses and Animals

H3N8 AIV GZA1 was isolated at the College of Veterinary Medicine, South China Agricultural University, Guangzhou, Guangdong, China. H3N8 AIV XJ47 was isolated and donated by the College of Animal Science and Technology, Shihezi University, Shihezi, Xinjiang, China [23]. The viruses were isolated from 10-day-old specific-pathogen-free (SPF) embryonic chicken eggs, as previously described [24]. Four-week-old SPF chickens were purchased from South China Biological Medicine (Guangzhou, China). Six-week-old female SPF BALB/c mice and six-week-old female SPF guinea pigs were purchased from Yancheng Biological (Guangzhou, China).

### 2.2. Sequence Analysis

According to the manufacturer’s instructions, the RNA extraction kit (Fastagen, Shanghai, China) was used to extract viral RNA from the allantoic-fluid-containing H3N8 strain. According to a previously published method [25], viral RNA was reverse transcribed into cDNA using Uni12 primers, and the genomic segments of the two AIV strains were amplified using specific primers. The PCR products were purified by a gel extraction kit (Omega, Guangzhou, China). The gel extraction products were sequenced by Sangon Biotechnology (Guangzhou, Guangdong, China). The sequence analysis was performed using Lasergene v7.1 (DNA Star Inc., Madison, WI, USA). BLAST searches of the two strains were performed using the GenBank database.

### 2.3. Animal Experiments

#### 2.3.1. Pathogenicity Studies in Chickens

Each group (*n* = 9) was inoculated intranasally and intraocularly with 10^6^ egg infective doses (EID_50_) of the indicated viruses in 100 μL. The contact groups (*n* = 6) were inoculated with 100 μL of phosphate-buffered saline (PBS), after inoculation with the virus, one day post-infection (dpi), and then one contact group was placed in the same cage as the inoculated group. The control group of six chickens was inoculated with 100 μL of PBS in the same manner. At 3 dpi, the heart, liver, spleen, lungs, kidneys, brain, trachea, thymus, pancreas, bursa of Fabricius, and cecum tonsils of the three inoculated chickens were collected after euthanasia. Viral detection in tissues and organs, the oropharynx, throat swabs, and cloacal swabs, as well as seroconversion studies, were conducted following a previously described method [26].

#### 2.3.2. Pathogenicity Studies in Mice

Each inoculated group (*n* = 18) was anesthetized with ether. Then, the mice of inoculated groups were inoculated intranasally with GZA1 or XJ47 at a dose of 10^6^ EID_50_ in 50 μL. The control group (*n* = 18) was inoculated with 50 μL of PBS after anesthesia. After infection, weight and mortality of mice were recorded daily for 14 days. Three mice in each group were euthanized at 3, 5, and 7 dpi, and the brain, lung, spleen, heart, liver, kidney, and nasal conchae were collected. Viral detection in tissues and organs was conducted following a previously described method [12]. At 3 dpi, the lungs of the inoculated group were placed in 4% paraformaldehyde and sent to Servicebio Technology Limited Company (Wuhan, China) for pathological analysis. Mice were humanely euthanized when they lost more than 30% of their original body weight.

#### 2.3.3. Pathogenicity Studies in Guinea Pigs

Each inoculated group (*n* = 3) was anesthetized with ether. Then, the guinea pigs of inoculated groups were inoculated intranasally with GZA1 or XJ47 at a dose of 10^6^ EID_50_ in 100 μL. The contact groups (*n* = 3) were inoculated with 100 μL of PBS at 1 dpi, and then each of the contact groups was placed in the same cage as the inoculated group, respectively. Three guinea pigs of the control group were inoculated with 100 μL of PBS in the same manner. The nasal cavity of each guinea pig was washed with 1 mL of PBS at 2, 4, 6, 8, and 10 dpi. Nasal washing samples were treated with 100 U/mL of penicillin and titrated in SPF embryonic chicken eggs, as previously described [26].

#### 2.3.4. Adaptation of the Virus to Mice

The two H3N8 AIV-adapted strains were designated as P12-GZA1 and P12-XJ47 after passaging 12 generations in mice, as described previously [27].

#### 2.3.5. Pathogenicity Studies of Adapted Strains

Pathogenicity studies of P12-GZ or P12-XJ in mice were performed using the method described in Section 2.3.2.

### 2.4. Sequence Analysis of Adapted Strains

The sequence analysis of P12-GZA1 and P12-XJ47 followed the method described in Section 2.2.

### 2.5. Statistical Analysis

GraphPad Prism 7.0 software (GraphPad Software Inc., San Diego, CA, USA) was used for the statistical analyses.

## 3. Results

### 3.1. Sequence Analysis of the Two H3N8 AIV Strains Isolated from Wild Birds

BLAST searches in the GenBank database showed that the GZA1 and XJ47 strains share high nucleotide homology with different AIV subtypes isolated from wild birds in Asian countries (Table 1). This genetic relationship indicates that GZA1 might have originated from reassortment between the native Chinese strains and different subtype strains from other Asian countries. The *HA*, *NA*, and *PB1* genes of GZA1 were highly homologous to the strains isolated from China. The other five genomic segments were closely related to the different subtypes of strains isolated in South Korea, Mongolia, and Thailand (Table 1). This genetic relationship indicates that GZA1 might originate from reassortment among the native Chinese strains with different subtype strains from other Asian countries. The surface genes *HA* and *NA* of XJ47 both showed high sequence similarity with those of A/duck/Mongolia/173/2015 (Table 1). Furthermore, analysis of the internal genes of XJ47 showed that it might have been a reassortant of different subtypes of strains carried by wild birds from Mongolia and several Asian countries to Xinjiang Province through long-distance migration.

The amino acid sequence of the HA protein cleavage site of the two H3N8 strains was PEKQT↓RGL, which is only one basic amino acid, consistent with the molecular characteristics of low pathogenic avian influenza viruses (LPAIVs). Among the HA fragments, both GZA1 and XJ47 have a T160A amino acid mutation, which has been reported to enhance binding to human α-2,6-linked sialic acid receptors [14]. Moreover, there are mutations D and A at position 30 and 215 of M1 in both strains, which can enhance virulence in mice [28]. GZA1 has a P42S mutation in the NS1 protein, which could potentially increase its virulence in mice (Table 2) [21].

### 3.2. Pathogenicity of the Two H3N8 Strains in Chickens

After infection with the GZA1 or XJ47 strain, no disease or death occurred among the SPF chickens during the 14 days. No virus shedding was detected in throat swabs and cloacal swabs collected at 3, 5, 7, and 9 dpi. In addition, only the XJ47 strain efficiently replicated in every collected tissue and organ (Table S1).

At 14 dpi, the antibody titers of the two inoculated groups and the XJ47 strain contact group were positive (Table S2). However, the GZA1 strain did not spread horizontally in the chickens (Table S2). Compared with the XJ47 strain, the GZA1 strain showed lower pathogenicity in chickens, which may be due to mutations in the HA and NS1 proteins of the GZA1 strain that are the result of adaptation to humans and mice (Table 2). Therefore, the pathogenicity of the two H3N8 strains in mice needs to be further determined.

### 3.3. Pathogenicity of the Two H3N8 Strains in Mice

**Figure 1:**
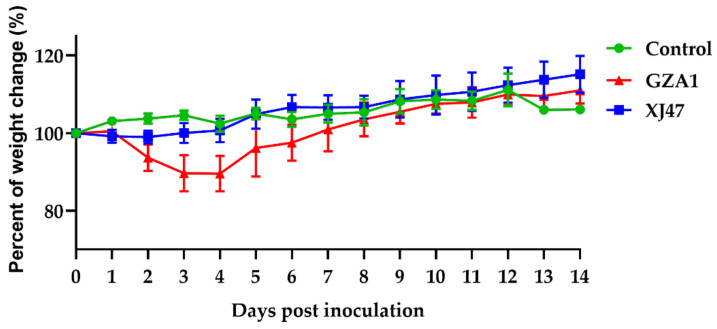
Daily weight changes of mice were recorded within 14 days after infection with the GZA1 and XJ47 strains. Values represent the mean percentage ± standard deviation (SD) relative to the original body weight of the mice.

No clinical symptoms were observed and no death occurred during the 2-week observation period after infection with the two strains. Compared with the control group, mice inoculated with the GZA1 strain showed a weight loss of approximately 10% at 2 dpi, which began to increase at 5 dpi (Figure 1). Mice infected with the XJ47 strain at 1–3 dpi only showed slow weight gain (Figure 1). This indicates that the GZA1 strain is more adapted to mice.

The GZA1 strain replicated efficiently in the heart, liver, lung, kidney, brain, and nasal conchae, and most effectively in the lung and nasal conchae (Table 3). The XJ47 strain replicated efficiently in all collected tissues and organs except for the kidney (Table 3). The two strains are thus suitable for efficient replication in mice, especially in the lungs and nasal conchae. The highest virus titers in the lung and nasal conchae of mice infected with the GZA1 strain were 3.08 log_10_ EID_50_/100 μL and 3.00 log_10_ EID_50_/100 μL, while those of mice infected with the XJ47 strain were 3.94 log_10_ EID_50_/100 μL and 3.58 log_10_ EID_50_/100 μL at 5 dpi (Table 3).

**Figure 2:**
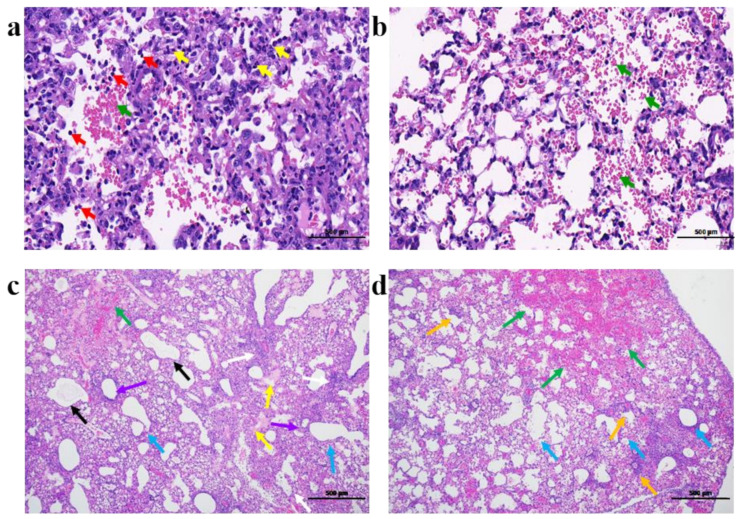
Pathological sections of mice lungs after infection with the four AIV strains: (**a**) GZA1: inflammatory cell exudation (red arrow), local bleeding (green arrow), and a large amount of inflammatory cell infiltration on the alveolar wall (orange arrow); (**b**) XJ47: local bleeding (green arrow); (**c**) P12-GZA1: eosinophilic fibrin exudation in the alveoli (black arrow), hyaline membrane formation (yellow arrow), small-scale hemorrhage (green arrow), alveolar cystic dilatation (blue arrow), incomplete bronchial epithelium, nucleolar clear and cytoplasm alkaline in epithelial cells (purple arrow), and infiltration of inflammatory cells (white arrow); (**d**) P12-XJ47: intra-alveolar hemorrhage (green arrow), focal infiltration of perivascular inflammatory cells (orange arrow), and alveolar cystic dilatation (blue arrow).

Inflammatory cell exudation, accompanied by local bleeding, and a large amount of inflammatory cell infiltration on the alveolar wall could be seen in a large area in the mouse lung after infection with GZA1 (Figure 2a). Mice infected with XJ47 showed only a small amount of hemorrhage in the lung tissue (Figure 2b).

### 3.4. Pathogenicity of Two H3N8 Strains in Guinea Pigs

The virus titers of guinea pig nasal washes were detected, and it was found that the two H3N8 strains could effectively replicate in guinea pigs.

**Figure 3:**
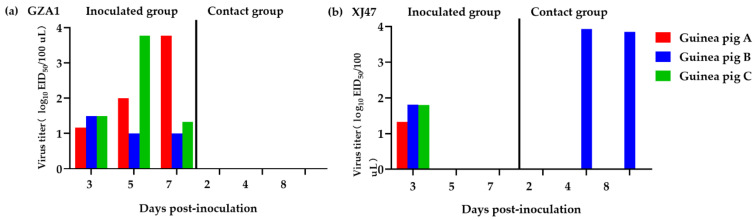
Virus titers of guinea pig nasal washes after infection with the GZA1 and XJ47 strains: (**a**) virus shedding of guinea pig after infection with the GZA1 strain; (**b**) virus shedding of guinea pig after infection with the XJ47 strain.

Virus shedding was detected in the GZA1 strain-infected group up to 3.77 log_10_ EID_50_/100 μL; however, virus shedding was not detected in any of the GZA1 contact guinea pigs (Figure 3a).

At 3 dpi, the XJ47 strain-infected group showed a small amount of virus shedding. Though only one guinea pig in the XJ47 strain contact group showed virus shedding, the virus titer was as high as 3.93 log_10_ EID_50_/100 μL (Figure 3b). This indicates that the GZA1 and XJ47 strains can spread horizontally in guinea pigs.

### 3.5. Molecular Characteristics of the Two Adapted Strains

After 12 passages, sequencing results show that multiple amino acid sites were mutated in both strains (Table 4). Among them, the P12-GZA1 adaptive strain mutated at the T235I, D408E, N448S, and D701N sites of the PB2 protein, the T413I and V609I sites of the PB1 protein, the T97I and P534S sites of the PA protein, and the A27V site of the NP protein. It has been reported that the D701N mutation of the PB2 protein can promote replication of the influenza virus in mammals and enhance its pathogenicity [19,29]. The mutation T97I in PA was also found in the P12-XJ47 strain, and it has been reported that the T97I could enhance the pathogenicity of H6N1 AIV [30]. Additional mutations occurred at the R17C and T35A of PB2 in the P12-XJ47 strain. The enhanced pathogenicity of the adaptive strain in mice may be related to mutations at these sites.

### 3.6. Pathogenicity of the Two Adapted Strains in Mice

**Figure 4:**
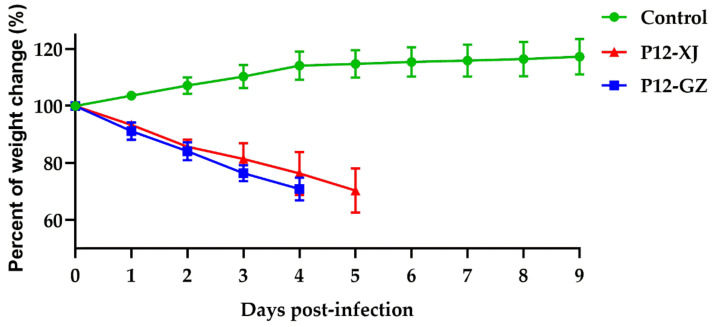
Daily weight changes of mice were recorded within 14 days after infection with the P12-GZA1- and P12-XJ47-adapted strains. Values represent the mean percentage ± SD relative to the original body weight of the mice.

To study the pathogenic changes of the two H3N8 isolates after transmission in mice, the pathogenicity of the two H3N8 strains in mice after adaptation was also evaluated. The adapted strains, P12-GZA1 and P12-XJ47, showed higher virulence in mice (Figure 4). At 2 dpi, the mice in the two inoculated groups showed obvious clinical symptoms such as depression, shortness of breath, trembling, and significantly reduced drinking of water and eating. In the P12-GZA1-inoculated group, three mice died at 3 dpi, and the others at 4 dpi. All mice infected with P12-XJ47 lost more than 30% of their initial body weight at 5 dpi, which was recorded as death.

Compared with the wild-type strain, the pathological changes in mouse lungs caused by the adapted strains P12-GZA1 and P12-XJ47 were more serious. The lungs of mice infected with P12-GZA1 exhibited more eosinophilic fibrin exudation in the alveoli, hyaline membrane formation, small-scale hemorrhage, alveolar cystic dilatation, extensive incomplete bronchial epithelium, large epithelial nucleus, clear nucleolus, basophilic cytoplasm, and small focal infiltration of inflammatory cells around many blood vessels (Figure 2c). There was more intra-alveolar hemorrhage, focal infiltration of perivascular inflammatory cells, and alveolar cystic dilatation in the lung tissues of mice infected with P12-XJ47 (Figure 2d).

## 4. Discussion

In this study, two H3N8 AIV strains isolated from wild birds were recombinant from different subtypes of AIVs. In HA and M1, both strains have mutations that can increase the virulence of mice, while the GZA1 strain has an additional mutation in NS1. Two wild-type strains replicated effectively in mice and guinea pigs. After 12 passages, the adapted strains caused more weight loss and death in mice. Several mutations were found in the adapted strains, and D701N in PB2 had been reported. In PA, the substitution T97I was found in both adapted strains, P12-GZA1 and P12-XJ47.

Ducks and wild birds have been frequently reported to play pivotal roles as reservoirs of LPAIVs. During a 2-year monitoring period, wild ducks raised in captivity served as monitoring sentinels for AIVs by being infected with four kinds of Eurasian-type AIVs, namely H6N8, H3N2, H2N3, and H3N8 [31]. In a 2-year migratory bird monitoring study in Liaoning Province, China, 27 strains of AIVs were isolated and divided into seven different subtypes, of which two were H3N8 [32]. Due to the migration of wild birds and live poultry trade activities, the viral recombination of different subtypes of AIVs in different regions might occur. In December 2016, a HPAIV H5N6 strain with a Japanese duck-origin H3N8 strain-derived *PA* gene was isolated at a local live poultry market in northern Vietnam [33]. A novel reassortant H3N8 avian influenza virus originating from North America and Eurasia was isolated from ducks at a live poultry market in Foshan City, Guangdong Province, China [34]. AIVs are known to be potentially infectious to humans and other mammals, even without adaptation [35,36]; for example, the H3N8 AIV from wild aquatic birds was able to cross the species barrier and establish successful infections in swine [37]. Therefore, the prevalence of the cross-species transmission of the H3N8 AIV in wild birds needs to be monitored.

Both adapted strains have the substitution T97I of the PA protein, which has been reported to potentially enhance polymerase activity, and to make the H6N6 AIV pathogenic to mice. In recent years, studies have shown that PA plays an important role in overcoming the host-species barrier, and facilitating the host adaptation of AIVs [38]. One earlier study found that PA of H1N1/2009 origin could increase the pathogenicity of the H9N2 AIV in mice, and the polymerase activity of influenza viruses in mammalian cell lines [39]. Another study showed that the amino acid residues 85I, 186S, and 336M from the PA protein derived from H1N1 could enhance the polymerase activity of the H3N2 AIV in mammalian cell lines, with 336M significantly improving the pathogenicity of the H3N2 virus in mice [40]. Another study determined that replacing I with R at position 353 of the PA protein contributed to the high virulence of chicken-origin H5N1 in mice [41]. The substitution T552S in the PA protein has been shown to increase the polymerase activity of AIVs, and their pathogenicity in mice [42].

The P12-GZA1 strain has the additional mutation D701N in the polymerase PB2 protein. A study reported that D701N in PB2 could enhance the transcription and replication activity of the H7N7 avian influenza virus strain in mammalian cells by enhancing the binding of the strain to importin-α1 [43]. In addition, D701N in PB2 has been found in a lot of IAVs, such as the H1N1 swine influenza virus (SIV) [44], the H3N8 AIV isolated from a grey seal [45], and the human H7N9 AIV [46]. Further study reported that D701N contributed to the pathogenicity of H1N1 SIV [47] and H5N1 AIV in mice [48].

In summary, our study provides two adapted H3N8 AIV strains to mice, and identifies several mutations that may increase the pathogenicity of H3N8 in mice.

## Figures and Tables

**Table 1:** Nucleotide sequences with the highest homology to GZA1 and XJ47 in the GenBank database.

Strain	Gene	Virus	GenBank Accession No.	Subtype	Identity (%)
GZA1	*HA*	A/Black-winged_curlew/China/CZ355/2019	MT835184	H3N8	99.04
	*NA*	A/baikal_teal/China/SH13(3)/2016	MT835170	H3N8	98.66
	*PB2*	A/white-fronted goose/Korea/F56-3/2017	MH130143	H6N2	99.17
	*PB1*	A/mallard/Shanghai/NH011204/2018	MN049585	H12N5	99.38
	*PA*	A/duck/Mongolia/140/2015	MK978919	H10N2	99.02
	*NP*	A/duck/Thailand/NA02/2003	MN629280	H3N2	98.86
	*M*	A/hooded crane/Korea/1176/2016	KY402068	H1N1	99.47
	*NS1*	A/mallard/Korea/F94-16/2017	MH579392	H4N6	99.74
XJ47	*HA*	A/duck/Mongolia/173/2015	LC121300	H3N8	98.00
	*NA*	A/duck/Mongolia/173/2015	LC121300	H3N8	99.29
	*PB2*	A/environment/Bangladesh/42007/2019	MW466087	H7N7	99.08
	*PB1*	A/mallard/Ukraine/AN-223-13-01/2020	MW855994	H7N3	98.68
	*PA*	A/duck/Mongolia/210/2018	MW188572	H3N8	99.35
	*NP*	A/Falcated duck/South Korea/JB42-30/2020	MW493162	H9N2	98.66
	*M*	A/mallard/Korea/F94-16/2017	MH579392	H4N6	99.74
	*NS1*	A/migratory bird/India/1722760/2017	MK453340	H4N6	99.40

**Table 2:** Molecular characteristics of GZA1 and XJ47.

Protein	Function	Mutation	GZA1	XJ47
HA	Human-type receptor binding	T160A	A	A
PB2	Mammalian adaptation	591R/K ^a^	Q	Q
M1	Enhanced virulence in mice	N30D	D	D
		T215A	A	A
NS1	Enhanced virulence in mice	P42S	S	A

^a^ according to H3N8 subtype.

**Table 3:** Virus replication and titers in tissues and organs of the infected mice.

Strains	Dpi	Heart	Liver	Spleen	Lung	Kidney	Brain	Nasal Conchae
GZA1	3	1/3 ^a^	1/3	0/3	3/3	2/3	0/3	3/3
		2.75 ± 0 ^b^	1.25 ± 0	- ^c^	3.66 ± 0.11	1.75 ± 0.25	-	3.33 ± 0.59
	5	1/3	2/3	0/3	3/3	2/3	2/3	3/3
		1.25 ± 0	1.25 ± 0	-	3.94 ± 0.55	1.25 ± 0	1.25 ± 0	3.58 ± 0.12
	7	1/3	0/3	0/3	2/3	0/3	0/3	1/3
		2.25 ± 0	-	-	2.13 ± 0.88	-	-	2 ± 0
XJ47	3	0/3	0/3	0/3	3/3	0/3	1/3	3/3
		-	-	-	3 ± 0.35	-	1.25 ± 0	2.16 ± 0.66
	5	1/3	1/3	1/3	3/3	0/3	1/3	3/3
		1.25 ± 0	1.25 ± 0	1.25 ± 0	3.08 ± 0.51	-	1.25 ± 0	3 ± 0.4
	7	1/3	0/3	1/3	1/3	0/3	0/3	2/3
		2 ± 0	-	1.25 ± 0	1.25 ± 0	-	-	1.25 ± 0

^a^ Virus isolation ratios of the mice infected with AIV. ^b^ Values represent average virus titers (log_10_ EID_50_/100 μL) ± standard deviation (SD). ^c^ No virus shedding.

**Table 4:** Mutations in amino acid sites of two mice adaptation strains of the two H3N8 AIV strains.

Strain	Protein	Mutation
P12-GZA1	PB2	T235I, D408E, N448S, D701N ^a^
	PB1	T413I, V609I
	PA	T97I ^b^, P534S
	NP	A27V
P12-XJ47	PB2	R17C, T35A
	PA	T97I

^a^ The D701N site of the PB2 protein can promote adaptation of H5N1 to mice [29]. ^b^ The T97I site of the PA protein can promote adaptation of H6N1 to mice [30].

## Data Availability

The genomic data presented in this study are available from GenBank (accession numbers: ON287054-ON287061, ON287062-ON287069).

## Supplementary Materials

The following supporting information can be downloaded at https://www.mdpi.com/article/10.3390/v14051097/s1, Table S1: Virus replication in tissues and organs of the chickens in the inoculated groups; Table S2: Seroconversion numbers of AIV-infected chickens at 14 dpi.
