# Supplementary material for: Adaptation of Two Wild Bird-Origin H3N8 Avian Influenza Viruses to Mammalian Hosts
Source: Viruses. 2022 May 19;14(5):1097. doi: 10.3390/v14051097 (PMC9147613; doi:10.3390/v14051097)
Supplement: Supplementary file 1 [file viruses-14-01097-s001.zip › viruses-1720401-supplementary.pdf]

Table S1. Virus replication in tissues and organs of the chickens in the inoculated groups.

Strains	Heart	Liver	Spleen	Lung	Kidney	Brain	Trachea	Thymus	Pancreas	Bursa of Fabricius	Cecum Tonsil
GZA1	0/3	0/3	0/3	0/3	0/3	0/3	0/3	0/3	0/3	0/3	0/3
XJ47	2/3 [a]	2/3	2/3	3/3	1/3	1/3	2/3	1/3	1/3	2/3	2/3

[a] Positive numbers of inoculated embryonic chicken eggs.

Table S2. Seroconversion numbers of the AIV-infected chickens at 14 dpi.

Strains	Inoculated groups	Contact groups
GZA1	6/6	0/6
XJ47	5/6	3/3

[a] Positive numbers of inoculated embryonic chicken eggs.
